# A perspective on the assessment of the broad value of vaccinations

**DOI:** 10.1093/eurpub/ckac129.236

**Published:** 2022-10-25

**Authors:** C de Waure, GE Calabrò, W Ricciardi

**Affiliations:** 1 Department of Medicine and Surgery, University of Perugia, Perugia, Italy; 2 Department of Life Sciences and Public Health, Università Cattolica del Sacro Cuore, Rome, Italy

## Abstract

Vaccinations are considered a cost-effective public health intervention to control vaccine-preventable diseases and are commonly evaluated in respect to several domains, including efficacy, safety and cost-effectiveness, before being implemented nationwide. There are plenty of evidence of economic evaluations performed on single vaccines or vaccinations. They commonly relies on static or dynamic models and considers both direct and indirect costs, i.e., those linked to productivity loss, of vaccine-preventable diseases. Nevertheless, vaccinations are expected to provide also societal benefits that call to the development and implementation of methods to value them, also from an economic point of view. The scientific debate on the assessment of the broad value of vaccinations has pinpointed several relevant aspects that need to be paid attention in future evaluations. They include the antibiotic sparing effect of vaccination, their impact on antimicrobial resistance - which is a challenge of our days -, their effect on social cohesion, their role in avoiding the loss of school days and in ameliorating educational attainment. The economic evaluation of vaccinations need to be further developed in order to allow a quantitative exploitation of their broad value according to the aspects described above. As the experience is still scant, we need to work on several fronts, including capacity building, data production and sharing and new methods development.

